# Capsule endoscopy in Crohn's disease surveillance: A monocentric, retrospective analysis in Italy

**DOI:** 10.3389/fmedt.2022.1038087

**Published:** 2022-11-28

**Authors:** Carlo Calabrese, Dania Gelli, Fernando Rizzello, Paolo Gionchetti, Rafael Torrejon Torres, Rhodri Saunders, Jason Davis

**Affiliations:** ^1^ IBD Unit, IRCCS, Azienda Ospedaliero-Universitaria di Bologna, Bologna, Italy; ^2^ Alma Mater Studiorum, Università di Bologna, Bologna, Italy; ^3^ Coreva Scientific, Koenigswinter, Germany

**Keywords:** crohn's disease, capsule endoscopy, biological treatment, symptomatology, outcome, real-world data, retrospective analysis

## Abstract

**Background:**

Crohn's disease (CD) is a potentially debilitating condition that burdens Italian healthcare substantially. The symptomatic management relies on prompt therapy adjustment to reduce flares and follow-up diagnostic inputs to maximise remission. Capsule endoscopy (CE) has introduced advantages in CD diagnostics, allowing the direct inspection of the entire gastrointestinal mucosa. The diagnostic procedure is comparable in effort to standard ileocolonoscopy (IC) but requires no anaesthesia. Whether CE follow-up improves clinical outcomes remains to be defined.

**Objectives:**

To provide a preliminary evaluation of CE in terms of clinical outcomes with respect to the standard of care ileocolonoscopy/MRE in Italy.

**Methods:**

This retrospective analysis utilises anonymised, monocentric data from the S. Orsola-Malpighi Hospital IBD database in Bologna, Italy, collected between 1999 and 2019. Out of 421 adult patient records, 100 were included in the analysis (50 per arm, matched per demographic and clinical characteristics). The CE represented the intervention arm, whereas ileocolonoscopy/magnetic resonance enterography was the standard of care. The use of biologics, symptomatology course, and surgery were the outcomes.

**Results:**

The two techniques performed similarly overall. In general, no significant difference emerged in the use of biologics. The use of biologics appears reduced in the CE group, only in L4 patients after the first follow-up year. Similarly, surgery was seemingly less frequent among L4 patients in the CE group. No difference was found between groups in flare occurrence and duration. CE patients might have experienced longer and earlier first remissions, but no long-term difference persisted.

**Conclusions:**

The CE group showed an apparent reduction in biologics and surgery, limiting to L4 diagnoses. More extensive, prospective, multicentre, randomised studies must corroborate these preliminary findings.

## Introduction

Crohn's disease (CD) is a potentially debilitating, idiopathic chronic inflammatory disorder of the gastrointestinal tract (GI) (1) and a considerable burden on the Italian healthcare system. In 2009, the prevalence was estimated to be 81 (female) and 91 (male) cases per 100,000 inhabitants, with an incidence of 6.5 and 7.4, respectively (2). More recent estimates increased these figures to 9.4 cases per 100,000 inhabitants a year (3). In terms of healthcare resources, ∼17,000 CD admissions were registered in Italy in 2005, 2008, and 2011 (4). The annual cost of disease has been appraised at €15,000 per patient, with direct costs comprising treatments and hospitalisations accounting for 76% of the total (5, 6).

From the patients' perspective, CD has a detrimental impact on quality of life, scoring between 0.52 and 0.76 on the EuroQol 5D scale for severe and milder forms, respectively (7). Although any section of the GI can be affected, the main foci are localised at the colon and small bowel. The aetiology remains unclear, and therapy has historically been symptomatic (8, 9). Long-term consequences caused by uncontrolled inflammation include fibrotic strictures, enteric fistulae, and intestinal neoplasia (10). Early diagnosis, treatment of inflammation, iterative disease assessment, and therapy adjustment effectively contribute to achieving clinical and endoscopic remission (10–13). These measures are also associated with reduced complications, shorter hospitalisations, and diminished resort to steroids, biologics, and surgical procedures (12, 14–16).

The diagnosis and continued surveillance of CD have relied on markers such as C-reactive protein (CRP) and faecal calprotectin (fCal), instrumental assessment by cross-sectional imaging, and direct visual mucosal inspection by ileocolonoscopy (IC), depending on the disease location and comorbidities. However, molecular markers and cross-sectional imaging have limited efficacy: The global magnetic resonance index of activity (MaRIA) and fCal values correlate moderately with endoscopic activity and do not correlate with each other (17). One in three CD patients does not exhibit high CRP levels during full-blown disease (18, 19), and ileal CD is not correlated with calprotectin levels (20, 21). Currently, IC remains the gold standard for mucosal assessment. However, the procedure is invasive, involves sedation and allows access to only a modest segment of the terminal ileum, while most of the small bowel is not accessible. Imaging the upper sections of the ileum is viable only with complementary ultrasonography, magnetic resonance enterography (MRE), and computed tomography (22).

Direct imaging of the small bowel became possible with the introduction of capsule endoscopy (CE), currently included in the ECCO Guidelines recommendations (9, 13, 23, 24). CE permits the direct imaging of the entire GI mucosa in a single procedure and was found to be better accepted by patients than IC (25–27). A multicentre study determined that the sensitivity of CE is equivalent to IC and/or MRE in each segment of the lower GI (28). Regarding specificity, CE outperforms MRE in the small bowel and is comparable to IC in the terminal ileum and colon (28–31). CE was previously shown also to yield comparable diagnostics to other non-invasive techniques (32–35). This is attributed to CE's greater sensitivity to lesions or defects in the bowel mucosa below the detection threshold of alternative imaging techniques and its significantly deeper coverage of the small bowel length than IC alone (32).

The management of CD articulates through early symptomatic treatment with corticosteroids to achieve rapid remission and long-term maintenance therapy to prevent flares and reduce the risk of complications (10, 36, 37). The maintenance treatment comprises immunomodulators and biologics for preventing flares and complications (10, 38). Despite these notable pharmacological advances, surgery remains necessary to treat approximately two-thirds of CD cases (39, 40).

However, the practical clinical implications of CE diagnostics and follow-up in guiding the course of treatment and remission have yet to be comprehensively characterised, i.e., whether CE-based diagnostics and follow-up have any real, practical benefit compared to IC/MRE in CD management. From this perspective, this study aims to provide a preliminary, retrospective, and monocentric gauge of the impact of CE on clinical outcomes in Italy.

## Methods

### Patient data

Retrospective, single-centre data were extracted from the IBD registry of the Azienda Ospedaliero-Universitaria Policlinico S. Orsola-Malpighi Hospital, Bologna, Italy. Data included limited patients' demographics (age, sex, disease parameters, and diagnostic method) in 6-month intervals over a five-year follow-up. An IC/MRE diagnosis qualified as the standard of care (SOC), whereas CE (Medtronic PillCam™ SB2/PillCam2) was the intervention group. Diagnoses in both arms were grouped per location according to the Vienna classification (41). No patients in the SOC group underwent CE, neither at first diagnosis nor during follow-up. Endoscopies of the upper GI were not performed in L4 diagnoses in the SOC group. All SOC patients received MRE at least once a year.

Data were pseudonymised by the data controllers (CC and DG) before transmission to the data processor (Coreva Scientific) and handled according to the General Data Protection Regulation (GDPR). The Sant'Orsola-Malpighi Hospital Ethics Committee reviewed and approved the study (approbation number 173/2017/O/OssN).

### Patient selection

The analysis included CD patients aged 18 or older with at least five years of available follow-up data from entry in the database. Patients who entered the database before 1999, missing diagnosed CD location or initial CD activity index (CDAI), were excluded. Patients with a history or symptoms of suspected strictures were ineligible for inclusion in the CE group and assigned to SOC.

### Outcomes

The outcomes investigated were the incidence of surgery, evolution of symptomatology in terms of CDAI score, and biologics therapy (prescription, initiation, and duration). Symptomatology was classified as *none* for CDAI < 150, *mild* for 150 ≤ CDAI < 220, *moderate* for 220 ≤ CDAI < 450 and *severe* for CDAI ≥ 450. Remission was defined as two consecutive asymptomatic intervals (one year, CDAI < 150). A flare was defined as the recurrence of moderate or severe symptoms (CDAI ≥ 220) following at least an asymptomatic (CDAI < 150) or mildly symptomatic year (150 ≤ CDAI < 220). Gaps in the CDAI data were excluded as *missing data*. Prescription data did not include the specific medication consistently and, therefore, did thus not allow stratifying per type of biologics.

### Diagnosis

#### CE procedure

The preparation for CE included a clear liquid diet for 24 h plus 12 h fasting and receiving 2 L of polyethylene glycol (PEG) solution 2–8 h before capsule ingestion (PillCam SB2/PillCam2). An additional fluid bolus was given after 2 h from capsule ingestion to facilitate transit through the small bowel (SB). All images were reviewed using the RAPID 8 software (Given Imaging, Yokneam, Israel). SB was divided into three main segments: proximal, medial, and distal. Mucosal inflammation was quantified using the Lewis Score (LS) (42, 43). Using the software application to calculate the LS, the SB was automatically divided into equal thirds (tertiles). SB lesions were considered proximal if located in the upper two-thirds of the SB (first two tertiles of the CE) and had an LS ≥ 135. SB inflammatory activity was classified as mild (135 ≤ LS < 790) or moderate-to-severe (LS ≥ 790). Gastric transit time and SB transit time were collected from the CE studies. In the event of an incomplete study, SB transit time was calculated as 480 min minus gastric transit time. A CE study was defined as complete if the capsule reached the cecum. Cleanliness in SB scored from 1 to 3 (1, free of stool and debris; 2, some stool and debris; 3, full of stool and debris). A board-certified gastroenterologist (CC) read the capsule videos.

#### Cross-sectional imaging

Examinations were performed with a single oral contrast preparation consisting of 1500 ml PEG solution ingested gradually for 45 min. MRE was carried out with an Intera 1.5 T MR system with a 5-element Synbody coil (Philips Medical Systems, Eindhoven, The Netherlands). Patients were examined supine. The protocol contained the sequences Cor T2 (B-FFE; TR/TE, 4.1/2.0 milliseconds; flip angle, 60°; slice thickness, 5 mm; 224 matrix; FOV 400), and axial T1W (TR/TE, 7/3.4; flip angle, 15°; slice thickness, 4 mm; 208 matrix; FOV 375), with discontinuous breath-hold before and after contrast. Gadodiamid 0.1 mmol/kg (GE Healthcare, Medical Diagnostics, Oslo, Norway) was given intravenously, and hyoscinbutyl-bromide 20 mg (Buscopan; Boehringer Ingelheim, Basel, Switzerland) was administered to reduce peristalsis during the procedure. CTE was performed with a 64-slice CT system (Somatom Sensation; Siemens, Erlangen, Germany) using: CARE-Dose: on, 120 kV, up to 150 mA; rotation time, 0.5 s; pitch, 1.5; collimation, 0.6 mm; increment 2. Contrast-enhanced CT scanning in the portal phase was performed after intravenous 100 ml iomeprol (Iomeron; Bracco, Milan, Italy) 300 mg/ml using an automatic injector OptiVantage DH (Mallinckrodt, Cincinnati, OH) at an injection rate of 4 ml/s. Patients with a body weight greater than 80 kg received 150 ml iomeprol at the same injection rate. All images were evaluated by using an Impax PACS workstation (Agfa, Mortsel, Belgium) with two Coronis monitors (1,600 × 200 pixels) (Megapixels Diagnostic Display System; Barco, Kortrijk, Belgium). CST readers identified active CD by segmental mural hyperenhancement, mural stratification, increased density in perienteric fat, sinus tract, or fistula. Fibrofatty proliferation and luminal narrowing without hyperenhancement were considered to represent inactive CD. Small bowel distension was rated on a two-point scale: sufficient (≥50%, score 1) or poor (<50%, score 0) for each examined segment. The image quality was rated as good (diagnostic images without artefacts, score 3), sufficient (diagnostic images with artefacts, score 2), or poor (non-diagnostic images, score 1). A bowel wall of 4 mm or more measured perpendicular to the bowel surface was considered thickened. Small bowel stenosis was defined as a change in bowel calibre with dilatation of the proximal segment above 2.5 cm and/or a collapse of the distal segment. MRE was interpreted at the time of examination by two experienced gastrointestinal radiologists. All SOC patients received MRE at least once a year.

### Data analysis

Data were processed using R v4.0.2 without *a priori* power calculation. Medians and interquartile ranges are provided for demographic data and outcomes, where applicable. Inference testing was performed using non-parametric tests (Fisher's exact test, Wilcox's test). A group matching algorithm was run using the R “MatchIt” package v3.0.2. Matching was performed over sex, age, disease duration, disease location, initial CDAI score, surgery history, and gastrointestinal bleeding.

## Results

### Analysis population

After applying exclusion criteria to 421 patient records, 325 were included in the study: 270 were diagnosed by IC/MRE (SOC group) and 55 by CE (intervention, CE group). After matching the groups, fifty patients per arm were ultimately included in the analysis. There was no statistically significant distinction between the groups' demographics after matching (Table 1). Subsequent analyses, stratified per CD location, excluded the L2 group due to low numbers.

**Table 1:** Patient groups after matching.

Parameter\group	SOC (*N* = 50)	CE (*N* = 50)	*p*-value
Age, median [IQR]		32 [24, 44]	34.5 [24, 40]	0.87
Female, *n* (%)		17 (34.0%)	22 (44.0%)	0.41
Disease duration, median years [IQR]		5.5 [1, 11]	4 [0, 9]	0.46
Disease location, *n* (%)	L1	19 (38.0%)	18 (36.0%)	0.81
	L2	2 (4.0%)	3 (6.0%)	
	L3	19 (38.0%)	16 (32.0%)	
	L4	10 (20.0%)	13 (26.0%)	
Previous surgery, *n* (%)		36 (72.0%)	33 (66.0%)	0.67
Previous GI haemorrhage, *n* (%)		30 (60.0%)	28 (56.0%)	0.84
Baseline CDAI score, median [IQR]		208.5 [137, 266]	205.5 [139, 259]	0.98
Symptomatology, *n* (%)	None	15 (30.0%)	15 (30.0%)	0.58
	Mild	12 (24.0%)	14 (28.0%)	
	Moderate	23 (46.0%)	19 (38.0%)	
	Severe	0 (0.0%)	2 (4.0%)	

Disease location is according to the Vienna classification, and symptomatology is estimated according to the Crohn's disease activity index (CDAI) score. See methods for range definitions. Continuous variables are shown as medians and interquartile ranges [IQR]. Categorical variables are shown as patient counts with percentages of the total in each treatment group. *P*-values were calculated using Fisher's exact test. SOC, standard of care; CE, capsule endoscopy.

### Outcomes

**Figure 1:**
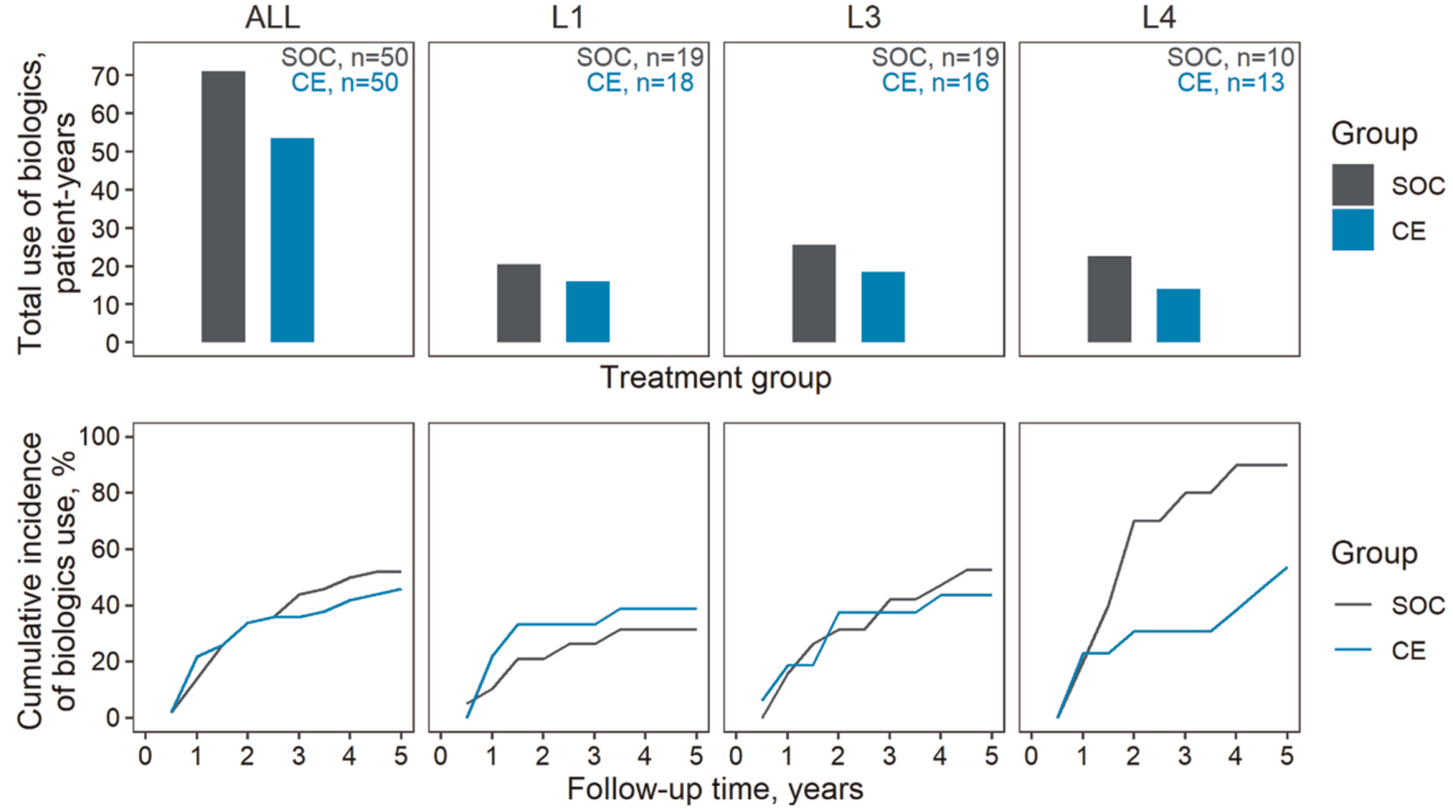
Biologics use. Biologics outcomes for the two management groups. Top, cumulative use of biologics through the five-year follow-up; bottom, cumulative incidence of biologics prescription. Results are stratified horizontally by diagnosed location: ALL, all diagnosed sites; L1, L3, and L4, disease location according to the Vienna classification. Note that L2 patients were excluded due to the low count.

The use of biologics in the two groups is summarised in Figure 1. Overall, the SOC group appeared to require more extensive use of biologics (71 patient years) than the CE group (53.5 patient years). However, this could be justified by the longer average disease duration in the SOC group (5.5 vs. 4.0 years); adjusting for disease duration rendered the difference marginal (CE, 73.5 patient years). Similar results emerged stratifying by the site of diagnosis. The two groups were comparable also in terms of initiation of biologics and time course throughout the follow-up, except for L4 diagnoses (22.5 patient years SOC vs. 14.0 patient years CE—Figure 1). Although L4 patients started therapy at the same time in both arms, the CE group seemed to make a reduced use of biologics after the first follow-up year (89.5% in SOC, 56.8% in CE at 5-year follow up).

**Figure 2:**
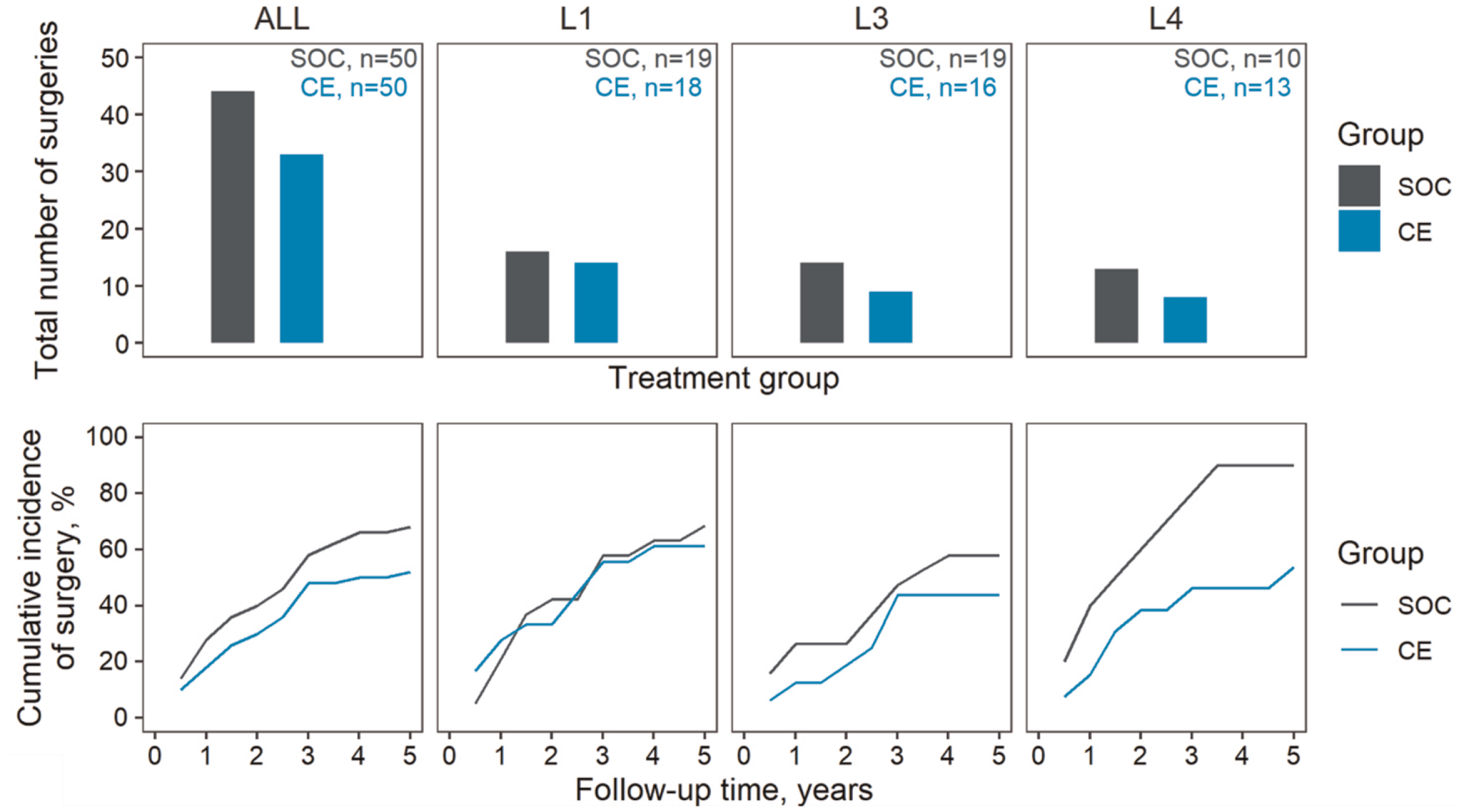
Surgery. Occurrence of surgery in the two groups. Top, cumulative number of surgeries through the five-year follow-up; bottom, cumulative incidence of surgery. Results are stratified horizontally by diagnosed location: ALL, all diagnosed sites; L1, L3, and L4 disease location according to the Vienna classification. Note that L2 patients were excluded due to the low count.

**Figure 3:**
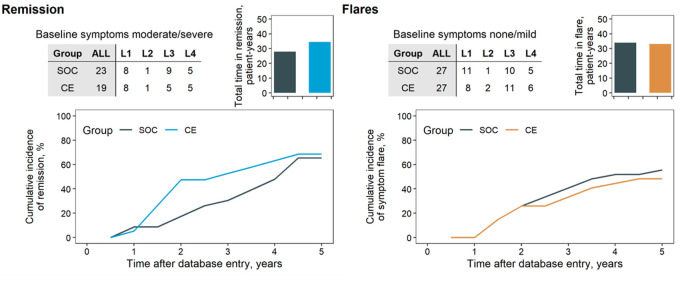
Cumulative incidence of remission and flares. Patients’ remission (top) and flare (bottom) analyses. Patients were divided by CDAI at baseline in moderate/severe symptoms at baseline for analysis of remission outcomes (top) and those with no or mild symptoms for analysis of flares (bottom). Analyses were not stratified per disease location because of the limited number of patients (tables, inset). Shaded columns indicate the total in each group in the analysis—four patients from the CE group due to missing CDAI score. Remission was defined as patients’ experiencing at least two consecutive intervals of no symptoms (CDAI score < 150). Flare was defined as the transition from a period of at least two consecutive intervals of no or mild symptoms (CDAI < 220) to at least one interval of moderate (CDAI score ≥ 220) or severe (CDAI score ≥ 450).

The reliance on surgery in the SOC and CE groups is summarised in Figure 2. Except for L1 diagnoses, the CE group apparently underwent less frequent surgery than SOC (67.3% in SOC, 53.7% in CE at five years). This effect was most distinct in L4 patients (88.1% in SOC, 53.4% in CE at year five). The course of symptoms (expressed as CDAI) between arms was compared depending on symptoms at baseline: Asymptomatic or mildly symptomatic patients at baseline were compared in terms of occurrence and duration of flares (Figure 3), whereas moderate-to-severe patients were compared regarding time in remission. This subdivision of the groups further thinned the numbers, making the comparison by diagnostic site impracticable. The total time in flare was comparable between the SOC and CE groups (34 patient years SOC vs. 33 patient years CE), as was the progression of flare incidence. Towards the follow-up conclusion, there was only a modest difference in the proportion of patients who had ever experienced a flare after a period of moderate or no symptoms (55.6% in SOC, 48.1% in CE). Patients in the CE group experienced a more prolonged cumulative remission than the SOC group (34.5 vs. 28.0 patient years). After the first year, CE patients achieved their first remission sooner. However, an equivalent fraction of patients had achieved at least one remission in both groups by the end of follow-up (68.4% CE, 65.2% SOC).

## Discussion

Capsule endoscopy has entered the field of IBD diagnoses and offers a competitive alternative to traditional IC/MRE imaging. Beyond indicators of increased sensitivity, technical performance, and improved patient acceptance, all of which are not to be overlooked, we questioned whether CE diagnostics alone brings tangible benefits to clinical outcomes. A substantial equivalence emerged between the two diagnostic approaches in the continued surveillance of CD patients. In general, neither the treatment choice nor the prognosis appeared to depend on the diagnostic approach. Nonetheless, despite the restrictions imposed by the unicentric, retrospective nature of the study and the small population size, some tendencies worthy of future investigations emerged. A preliminary, qualitative consideration concerns the apparent dichotomy in the course of biologics use among L4 patients. Although biologics were initiated at approximately the same time in the two arms, the cumulative incidence of biologics progressed slower among L4 patients in the CE group from the first year of follow-up. A similar reduction in the overall incidence of surgery is observed among L4 patients in the CE group throughout follow-up. A hypothesis to explain the decreased use of biologics and surgery in the CE group, notably among L4 patients, could be a superior clinical efficacy of CE in continued vigilance, i.e., CE might allow for a prompter and more flexible therapy remodulation (28), in particular in the medium-to-long term management of upper GI lesions. However, the data at hand and its limitations in statistical significance do not allow for numerically elaborating nor corroborating this observation's determinants nor discussing the role of cofounders, such as a history of flare-ups or smoking. The absence of upper GI endoscopies, as recommended by ECCO-ESGAR guidelines in 2018 for patients with CD with upper GI symptoms (44), may constitute an additional limitation of this study.

Any definitive conclusions necessitate a larger sample. Nonetheless, this hypothesis is in line with our recent experience in the proactive use of CE in CD patient management, including active CD (31) and evidence of proximal bowel CD as a poor prognostic factor for therapeutic escalation (45, 46). Ultimately, this analysis does not consider aspects for a comprehensive evaluation of the two techniques from the healthcare standpoint. Policymaking is heavily reliant on patient acceptance, procedural simplification and, not least, monetary considerations. While the literature supports the general findings on the clinical benefits of CE in the local context (28, 46, 47), the monetary aspects have yet to be quantified within the standards of the Italian healthcare system. In conclusion, we interpret these data qualitatively, wary of the existing constraints, and urge additional multicentre studies in Italy.

## Conclusions

In this retrospective, matched cohort analysis, we observed a reduced use of biologics and surgery in patients with L4 disease in the CE group. In addition, the CE group also showed greater incidence and duration of remission independent of disease location for patients symptomatic at baseline. More extensive, prospective, multicentre, randomised studies are required to corroborate these preliminary findings.

## Data Availability

The raw data supporting the conclusions of this article will be made available by the authors, without undue reservation.
